# Association between celiac and superior mesenteric arteries’ Doppler flow parameters and risk of necrotizing enterocolitis in preterm infants

**DOI:** 10.1177/19345798251377439

**Published:** 2025-09-13

**Authors:** Raef Qeretli, Abdalkarim Alnajjar, Nadya Ben Fadel

**Affiliations:** 1 27338 Department of Pediatrics, Children’s Hospital of Eastern Ontario (CHEO), Ottawa, Canada; 2 Department of Public Health Sciences, 4257 Queen’s University, Kingston, Canada; 3 Faculty of Medicine, University of Ottawa, Ottawa, ON

**Keywords:** necrotizing enterocolitis, preterm infants, superior mesenteric artery, Doppler ultrasound, intestinal perfusion, neonatal morbidity

## Abstract

**Background:**

Necrotizing enterocolitis (NEC) remains a significant cause of morbidity and mortality in preterm neonates. Identifying early markers of impaired intestinal perfusion may aid detecting an association with the development of NEC. This study aims to evaluate the role of Doppler ultrasound of the superior mesenteric artery (SMA) and celiac artery (CA) and its association with NEC in preterm neonates.

**Methods:**

We conducted a retrospective, single-center case–control study. Eligible infants were born at <29 weeks’ gestation; we excluded those with chromosomal abnormalities, major anomalies, and those without Doppler assessments. NEC cases (Bell stage ≥II) were matched to controls on gestational age and birth weight. We compared SMA and CA Doppler parameters—peak systolic velocity (PSV), end-diastolic velocity (EDV) of NEC and control infants obtained at the end of the first and between 2^nd^ and 3^rd^ weeks of life.

**Results:**

Among 44 preterm infants (NEC = 21; controls = 23), Doppler assessment in the 1^st^ week showed lower **CA** PSV in NEC versus controls (AMD = −27.7 cm/s [−53.58, −1.81]; *p* = 0.0371) after adjustment for PDA, birth weight, and gestational age. In weeks 2–3, and before NEC onset, NEC infants had lower **SMA** PSV (AMD = −35.7 cm/s [−68.5, −3.00]; *p* = 0.036) in models adjusted for PDA. No significant differences were found in CA parameters.

**Conclusions:**

Reduced CA PSV during the first week of life, and reduced SMA PSV prior to NEC onset reflects impaired splanchnic perfusion preceding NEC and may be useful to clinicians in stratifying neonates at a risk of developing NEC in advance.

## Introduction

Neonatal necrotizing enterocolitis (NEC) is one of the most frequent and life-threatening gastrointestinal emergencies in newborns.^1^ Its incidence varies widely by region and neonatal intensive care unit (NICU), ranging from 1 to 5% of NICU admissions (equivalent to fewer than 1 to approximately 3 cases per 1000 live births).^2^ Mortality rates likewise differ depending on birth weight, with estimates of 42% in infants weighing 501–750 g and approximately 15.9% in those weighing 1251–1500 g.^3^

Bell and colleagues introduced the first classification system for NEC in 1978,^4^ and it remains widely used today. In 1986, Michele C. Walsh and colleagues proposed a Modified Bell Staging, refining the original system by adding more stages and allowing better differentiation of disease severity.^5^ From a pathophysiological standpoint, compromised blood flow to the intestine is an important contributor to the development of NEC. The abdominal aorta’s major branches—the celiac artery (CA), superior mesenteric artery (SMA), and inferior mesenteric artery—play critical roles in bowel perfusion. The CA supplies the spleen and structures derived from the embryonic foregut,^6^ whereas the SMA is the principal blood supply to the small intestine and portions of the colon.^7^ Doppler ultrasound imaging of these arteries can thus provide valuable insights into splanchnic blood flow and the potential risk for NEC. Although relatively few studies have directly examined whether SMA and/or CA Doppler parameters predict NEC, several investigations indicate that early abnormalities in these flow parameters may correlate with adverse bowel outcomes. Some studies have demonstrated that neonates with abnormal Doppler flow in the first few days of life have been associated with both intestinal dysmotility^8^ and development of NEC.^9^ Coombs et al. reported a reduction in SMA blood flow velocity in neonates at risk of NEC during the first 4 days of life, suggesting compromised splanchnic circulation.^10^ Hashem et al. similarly found decreased peak systolic (PSV) and end-diastolic velocities (EDV) in the SMA among neonates with NEC.^11^ However, Kempley et al. observed elevated SMA blood flow velocity at the time NEC symptoms became apparent.^12^

The onset of NEC is inversely related to gestational age; neonates born before 28 weeks of gestation and those below 1000 gm tend to develop NEC around 22–25 days of life.^13,14^

In our NICU, standard practice involves screening infants born less than 29 weeks for patent ductus arteriosus (PDA) with echocardiography, during which SMA and CA blood flow is also routinely evaluated. Echocardiography, including Doppler flow assessment, is typically repeated based on PDA status and treatment plan, as well as the patient’s clinical status.

This study aims to evaluate the association between SMA and CA Doppler flow parameters and the subsequent development of NEC in neonates born before 29 weeks of gestation. Doppler measurements from infants who developed NEC were compared with those from a matched cohort without NEC to assess the potential for identifying infants at increased risk.

## Methods

### Ethics and confidentiality

This research protocol was formally approved by The Ottawa Hospital Research Ethics Board (REB) (Approval # 20220734-01H).

### Study design and setting

We performed a retrospective, single-center, case–control study in the NICU at The Ottawa Hospital-General Campus (TOH-GC), including admissions from January 1^st^ , 2018, to December 30^th^, 2022.

### Participants

Of the 343 NICU admissions in the study period, we considered only preterm infants born at ≤29 weeks’ gestation. We excluded infants with chromosomal abnormalities (e.g., trisomy 13 or 18) or major congenital anomalies, those with missing Doppler assessments, and those with significant congenital heart disease other than PDA, small ASD/PFO, or small VSD. NEC cases were selected if they were classified at Modified Bell’s stage IIA or higher. Controls were selected with no history of NEC or culture-positive sepsis and were matched to cases on gestational age and birth weight. The final study cohort consisted of 21 infants with NEC and 23 matched controls.

### Clinical care

To reduce confounding from physiologic factors known to affect intestinal blood flow in preterm infants, clinical care practices were standardized across groups: all infants received exclusive breast milk feeds with volumes per unit protocol; Doppler assessments were performed pre-prandially; and all infants received antenatal corticosteroids, maternal magnesium sulfate, and postnatal caffeine therapy. All infants diagnosed with NEC received medical management at diagnosis, with surgery reserved for cases meeting criteria for complicated NEC. For the purpose of this study, we define hemodynamically significant PDA as any PDA that needed medical or surgical intervention, as decided by our Neonatal hemodynamic team.

### Data sources and Doppler assessments

Eligible participants were identified through electronic health records in the hospital’s data warehouse. Doppler flow assessments were conducted as part of our PDA assessment guidelines with echocardiography performed by the Neonatal Hemodynamic Team by two expert physicians. According to the PDA assessment protocol, screening is conducted between 3 and 5 days of life, for infants with a gestational age of less than 26 weeks or earlier if clinical findings are present. For those with a gestational age ≥26 weeks and ≤29 weeks, screening occurs between days 5–7 of life, or earlier if clinically indicated. Echocardiography is repeated weekly during the 1^st^ month of life to follow up PDA closure. Data collected included, CA, SMA, and aortic flow parameters, including PSV, EDV, and resistive index (RI), as well as demographic and clinical characteristics. Small for Gestational age status (SGA) was defined based on the Fenton 2013 preterm growth chart, using a birth weight below the 10th percentile.^15^

### Outcomes and analyses

#### Primary outcome

The primary outcome measures were the CA and SMA Doppler parameters (PSV, EDV, RI) of NEC patients obtained in the first week of life, and in the week preceding NEC diagnosis (weeks 2–3 window), compared with matched controls without NEC during the same periods of time.

**Figure 1.**
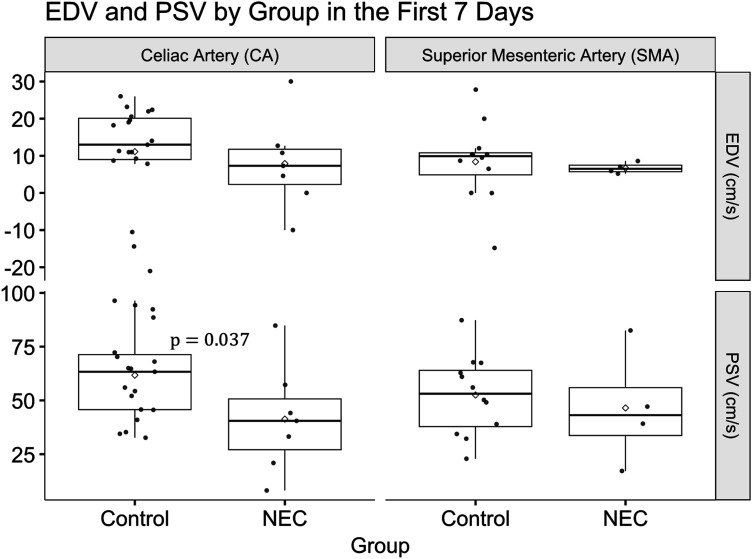
Boxplots illustrating EDV (cm/s) (top row) and PSV (cm/s) (bottom row) in the CA (left column) and SMA (right column) in the first 7 days of life. Dots represent individuals and diamonds represent means.

Table 2 presents first-week (≤ 7 days) measurements for all infants who subsequently developed NEC compared with controls, providing a baseline comparison between groups. At the time of measurements, no patient had developed NEC. Using linear regression, we controlled for PDA status, gestational age, and birth weight, factors which are known to affect blood flow. The distribution of Doppler parameters in Table 2 is shown in Figure 1.

**Figure 2.**
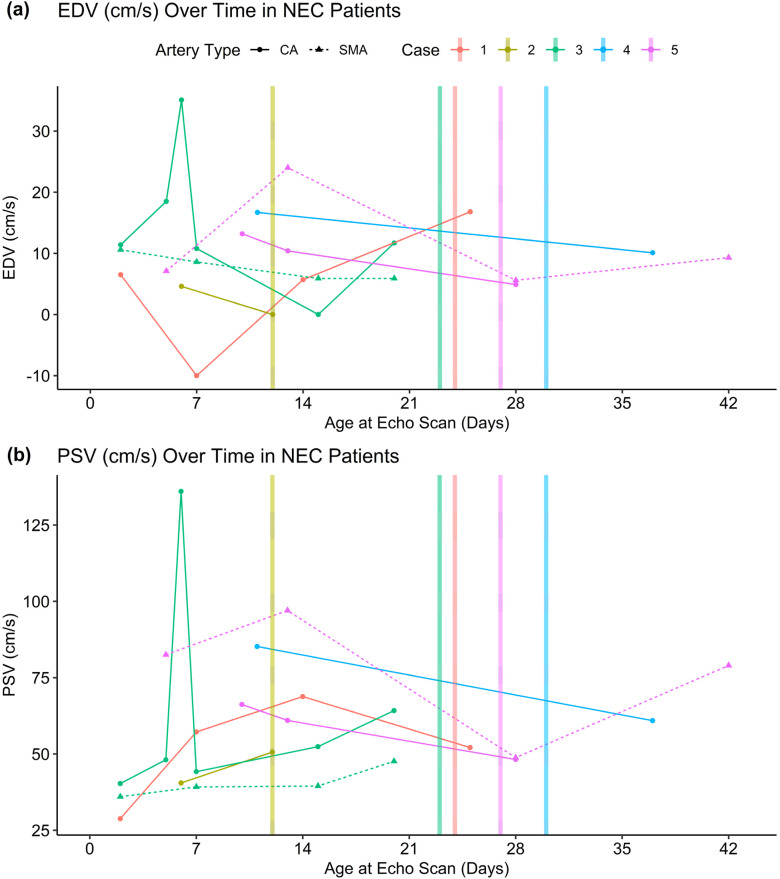
Time series of (a) EDV (cm/s) and (b) PSV (cm/s) in select NEC patients. Each patient is represented by a distinct color, and the onset of NEC is marked by a vertical line. Solid lines with circular markers indicate CA measurements, while dashed lines with triangular markers indicate SMA measurements.

Table 3 focuses on weeks 2–3, using the last ultrasound performed before NEC onset and comparing these values to controls during the same postnatal period. At the time of measurements, no patient had developed NEC. Using linear regression, we controlled for PDA status, but we opted not to control for gestational age or birth weight at this stage, since generally their effects on Doppler flow become less pronounced.^16^ The choice of this reference period was because this period corresponds to the typical timing when most preterm infants have achieved full enteral feeds and coincides with the highest incidence window for NEC.^17^ Figure 2 is a time-series plot of Doppler parameters to visualize the change in flow over time for NEC infants around the time of NEC development. 5 cases were included based on having enough data points to warrant inclusion in a time-series plot.

#### Secondary/descriptive outcomes

We also report the incidence of NEC among all NICU admissions during the study period; patient demographics and perinatal characteristics (Gestational age including SGA, birth weight, sex, mode of delivery); PDA presence, hemodynamic significance, and management (medical, surgical, conservative); age at NEC diagnosis; culture-positive sepsis at NEC suspicion; inotrope use related to NEC; and NEC management category (medical, surgical, medical + surgical, or palliative).

### Statistical analysis

Data were summarized using mean ± standard deviation (SD), median and range, or number of cases and percentages, as appropriate. Comparisons between study groups for numerical variables were conducted using Student’s *t*-test for independent samples when the data followed a normal distribution, and the Mann–Whitney *U* test when normality was not met. In cases of hypothesis testing (Tables 2 and 3), we used linear regression to extract the adjusted mean difference (AMD) between NEC and Control and 95% confidence intervals. Categorical data were analyzed using the Chi-square (*χ*^2^) test, or the exact test, where appropriate. All analyses were performed using R Statistical Software (v4.4.2;^18^). Graphs and data manipulation were conducted using base R and the “tidyverse” R package (v2.0.0;^19^).

## Results

### Demographic and clinical characteristics

A total of 44 preterm infants were included in this study: 23 in the control group and 21 in the NEC group. The key demographic and clinical characteristics are summarized in Table 1. The mean gestational age was significantly lower in the NEC group (25.6 ± 1.87 weeks) compared to the control group (26.8 ± 1.64 weeks, *p* = 0.025). Similarly, birth weight was significantly lower in the NEC group (705 ± 142 g) compared to the control group (922 ± 228 g, *p <* 0.001). The proportion of infants classified as SGA was higher in the NEC group (23.8%) compared to controls (8.7%). However, this difference was not statistically significant. No significant differences were observed in gender distribution or delivery mode between groups.

**Table 1.** Patient demographics and clinical variables.

	Control (N = 23)	NEC (N = 21)	*p*-value
Gestational age (weeks)			
Mean (SD)	26.8 (1.64)	25.6 (1.87)	0.0246
Median [min, max]	27.6 [23.0, 28.9]	25.1 [23.1, 28.7]	
Small for gestational age			
Yes	2 (8.7%)	5 (23.8%)	0.232
No	21 (91.3%)	16 (76.2%)	
Birth weight (grams)			
Mean (SD)	922 (228)	705 (142)	<0.001
Median [min, max]	910 [580, 1280]	680 [482, 1040]	
Gender			
Female	8 (34.8%)	9 (42.9%)	0.811
Male	15 (65.2%)	12 (57.1%)	
Delivery mode			
Cesarean section	10 (43.5%)	10 (47.6%)	1
Vaginal delivery	13 (56.5%)	11 (52.4%)	
NEC outcome			
Medical	NA	11 (52.4%)	NA
Medical and surgical		2 (9.5%)	
Palliation		3 (14.3%)	
Surgical		5 (23.8%)	
Patent ductus arteriosus (PDA)			
No	7 (30.4%)	3 (14.3%)	0.287
Yes	16 (69.6%)	18 (85.7%)	
PDA severity			
Hemodynamically significant	9 (39.1%)	13 (61.9%)	0.5397
Non-hemodynamically significant	7 (30.4%)	5 (23.8%)	
No PDA	7 (30.4%)	3 (14.3%)	
PDA management			
Medical	6 (26.1%)	11 (52.4%)	0.009
Surgical	3 (13.0%)	1 (4.8%)	
Conservative	0 (0%)	3 (14.3%)	
Medical and surgical	0 (0%)	2 (9.5%)	
PDA didn’t require management	7 (30.4%)	1 (4.8%)	
No PDA (N/A)	7 (30.4%)	3 (14.3%)	
Inotrope use (following NEC diagnosis)			
No	NA	18 (85.7%)	NA
Yes		3 (14.3%)	
Type of inotrope used			
Dobutamine and dopamine	NA	1 (4.8%)	NA
Norepinephrine		1 (4.8%)	
Norepinephrine, dobutamine, and vasopressin		1 (4.8%)	
None		18 (85.7%)	
Age at NEC diagnosis (days)			
Mean (SD)	NA	20.3 (16.1)	NA
Median [Min, Q1, Q3, Max]		14 [7, 10, 24, 69]	
Culture-positive sepsis at the time of NEC			
No	NA	16 (76.2%)	NA
Yes		5 (23.8%)	

Despite not being statistically significant, PDA was more common among NEC infants (85.7%) than controls (69.6%), and 61.9% of NEC infants had hemodynamically significant PDA compared with 39.1% of controls. However, management approaches differed notably between groups; medical management was significantly more frequent in the NEC group (52.4% vs 26.1%, p = 0.009). Other PDA management strategies, including surgical or conservative approaches, were less common overall.

Among NEC infants, the mean age at diagnosis was 20.3 ± 16.1 days, with a median of 14 days [IQR: 10–24, range: 7–69]. At the time of NEC diagnosis, 23.8% of infants had culture-positive sepsis. Inotrope support following NEC diagnosis was required in 14.3% of cases, with varying regimens, most commonly norepinephrine-based combinations. Outcomes among NEC infants included medical management alone in 52.4%, surgical management in 23.8%, combined medical and surgical intervention in 9.5%, and palliation in 14.3%.

### Doppler flow parameters in the 1^st^ week of life

In the first week of life, infants who went on to develop NEC demonstrated lower mean CA PSV (41.3 ± 24.9 cm/s) compared with controls (61.7 ± 20.5 cm/s), with an adjusted mean difference of −27.7 (95% CI: −53.6, −1.8) after controlling for PDA status, birth weight, and gestational age. No significant differences were observed between groups for EDV or RI in either the CA or SMA, or in the SMA PSV. Similarly, the age at echocardiography did not differ significantly between NEC and control infants (Table 2). Boxplots in Figure 1 illustrate the distribution of Doppler flow parameters in the CA and SMA for both control and NEC groups. The Boxplots highlights the lower PSV observed in CA among NEC cases, consistent with the statistically significant finding in Table 2.

**Table 2.** Doppler flow parameters in NEC vs control infants (first week of life).

	Celiac Artery			Superior Mesenteric Artery^a^	
	Control (N = 19)	NEC (N = 7)	AMD [95% C.I]	Control (N = 12)	NEC (N = 4)
PSV (cm/s)					
Mean (SD)	61.7 (20.5)	41.3 (24.9)	−**27.7 [**−**53.58,** −**1.81]**	52.5 (18.3)	46.5 (27.1)
EDV (cm/s)					
Mean (SD)	11.1 (13.1)	7.91 (12.3)	NS	8.40 (10.5)	6.70 (1.49)
RI					
Mean (SD)	0.783 (0.229)	0.739 (0.334)	NS	0.833 (0.167)	0.815 (0.0940)
Age at echo scan (days)					
Mean (SD)	4.74 (1.97)	5.57 (2.15)	NS	4.25 (1.96)	5.25 (1.71)

NS = Not statistically significant.

^a^No significant differences found in adjusted and unadjusted analyses.

### Doppler flow parameters in weeks 2 – 3

In weeks 2 and 3 of life and prior to NEC onset, CA flow parameters did not differ significantly between groups. However, SMA PSV was significantly reduced in NEC infants (44.7 ± 8.0 cm/s) compared with controls (78.2 ± 25.2 cm/s), with an adjusted mean difference of −35.7 (95% CI: −68.5, −3.0) after controlling for PDA status. End-diastolic velocity did not differ significantly between groups. The age at echocardiography was similar between groups (Table 3).

**Table 3.** Doppler flow parameters in NEC vs control infants in weeks 2 and 3 and before the onset of NEC.

	Celiac Artery^a^		Superior mesenteric artery		
	Control (N = 15)	NEC (N = 7)	Control (N = 8)	NEC (N = 4)	AMD [95% C.I.]
PSV (cm/s)					
Mean (SD)	67.8 (23.0)	64.4 (10.7)	78.2 (25.2)	44.7 (8.03)	**−35.74 [-68.49, −2.99]**
EDV (cm/s)					
Mean (SD)	4.36 (12.9)	10.7 (10.4)	−0.825 (13.7)	8.95 (4.11)	NS
RI					
Mean (SD)	0.831 (0.254)	0.850 (0.130)	0.975 (0.162)	0.795 (0.0926)	**−0.22 [-0.42, −0.03]**
Age at NEC diagnosis (days)					
Mean (SD)	NA	17.7 (6.32)	NA	18.0 (6.38)	NA
Median [min, max]		19.0 [9.00, 24.0]		18.0 [12.0, 24.0]	
Age at echo scan (days)					
Mean (SD)	14.9 (5.00)	13.0 (4.24)	13.5 (6.09)	12.3 (6.13)	NS
Median [min, max]	15.0 [7.00, 22.0]	14.0 [7.00, 20.0]	12.5 [7.00, 22.0]	11.5 [6.00, 20.0]	

NS = Not statistically significant.

Unadjusted analyses: In univariate analyses using linear regression, SMA PSV was significantly lower in the NEC group compared with the control group (*p* = 0.0295; difference = −33.50 [95% CI: −62.91, −4.09]).

^a^No significant differences found in adjusted and unadjusted analyses.

Figure 2 presents a time-series plot of EDV (panel a) and PSV (panel b) of select NEC patients. The vertical dashed line marks the onset of NEC. Each patient is represented by a different color, with solid lines indicating measurements from CA and dashed lines indicating measurements from the SMA. The figure provides a visualization of the change over time, with particular interest to changes around the onset of NEC. In certain cases, there was a progressive decline in both EDV and PSV in both arteries before the onset of NEC. While variations were observed among individual patients, the overall trend suggests a reduction in blood flow velocities in the days before NEC diagnosis.

## Discussion

Our study examined whether Doppler flow parameters of the SMA and CA are associated with subsequent necrotizing enterocolitis (NEC) in infants born before 29 weeks’ gestation. Despite efforts to match cases and controls on gestational age and birth weight, NEC cases remained younger and smaller; an observation consistent with prior literature linking lower birth weight and gestational age to NEC risk.^13,14,17^ These baseline differences highlight the practical difficulty of achieving perfect comparability between NEC cases and controls and raise the possibility of residual confounding. Notably, the higher prevalence of hemodynamically significant PDA and the greater need for medical management among NEC cases align with a pathophysiologic model in which compromised mesenteric perfusion contributes to NEC development. Together, these findings support the biological plausibility that alterations in mesenteric blood flow precede or accompany the cascade leading to NEC.

Two temporally distinct Doppler findings emerged. First, within the first week of life, well before NEC onset. NEC cases had lower CA PSV; a finding that persisted after adjustment for PDA, gestational age, and birth weight. Second, in the second/third week closer to the clinical event, SMA PSV was lower in NEC infants compared with controls, after accounting for PDA status. Taken together, these results suggest that systolic forward flow particularly in the SMA territory most relevant to NEC, may be the more sensitive Doppler marker in the prodromal window, whereas early CA changes in week 1 could signal a broader systemic or ductal-steal–related perfusion vulnerability.

The longitudinal plots (Figure 2) complement the groupwise analyses by illustrating, in several NEC cases, a gradual decline in PSV and EDV in the days preceding diagnosis. Although patient-level trajectories were heterogeneous and points were limited, the recurrent pattern of pre-NEC deceleration in mesenteric velocities supports the biological plausibility that evolving perfusion compromise contributes to pathogenesis rather than simply accompanying it. Importantly, the ages at echocardiography were similar between groups in both time windows (Tables 2 and 3), reducing concern that timing differences alone explain these our findings.

Clinically, these findings point to two pragmatic implications. First, repeated Doppler assessment may be more informative than a single assessment, with particular attention to SMA PSV trends in the second or third week of postnatal age. Second, when interpreting Doppler indices for NEC risk stratification, potential confounders should be considered. Our adjusted analyses address some of this concern, but residual confounding remains possible given the modest sample and wide confidence intervals. Future studies with larger samples and standardized, serial pre-prandial scans could test whether prespecified velocity thresholds or trajectory-based alerts improve early identification of infants at highest risk.

Previous research has indicated that abnormalities in splanchnic circulation, such as impaired mesenteric perfusion, may contribute to NEC.^9–11^ Other studies have reported mixed findings regarding SMA blood flow. For example, Coombs et al. observed that SMA PSV increased over the first 4 days of life in healthy neonates but was delayed in neonates at risk for NEC.^10,12,20^ Conversely, higher SMA (and CA) velocities reported at symptom onset likely reflect acute hyperemia and inflammation rather than a predictive signal.^12,20^

Our study differs from these by focusing on Doppler measurements obtained before clinical onset, where we observed a lower CA PSV in the first week of life and lower SMA PSV prior to NEC onset, suggesting possible hypoperfusion prior to disease manifestation.

A study by Yue et al. investigated the predictive value of early Doppler ultrasound of SMA in preterm neonates.^21^ They found significantly higher PSV in infants who later developed NEC. These findings suggest that abnormal SMA blood flow within the first 3 days of life may serve as an early predictor of NEC.

Additional studies have provided insights into splanchnic blood flow abnormalities and NEC. Murdoch et al. demonstrated that reduced SMA flow velocity within the first 24 h of life was associated with an increased risk of NEC.^9^ Hashem et al. found that preterm infants with neonatal sepsis and at risk for NEC had reduced SMA and CA flow velocities, further suggesting that systemic inflammatory responses may contribute to altered gut perfusion.^11^ These findings reinforce the hypothesis that disturbances in splanchnic blood flow may contribute to NEC pathogenesis and highlight the need for serial Doppler monitoring in high-risk neonates.

We acknowledge that our study has few limitations. First, the sample was modest (44 infants; 21 with NEC), which reduces statistical power, yields wide confidence intervals, and limits stratified or fully adjusted modeling (e.g., by NEC timing, stage, SGA/AGA, etc.) without overfitting. Second, residual confounding is possible despite adjustment for gestational age, birth weight, and PDA status. Third, echocardiography was obtained for clinical indications rather than a fixed research schedule; although ages at scan were similar between groups, scan timing and number of repeats varied, and Doppler measures are susceptible to operator and physiologic variability. While we had two trained and well experienced physicians perform Echocardiography and Doppler assessment of the intestinal vasculatures, the interobserver variability couldn’t be excluded. Relatedly, the time-series figure illustrates only the subset of NEC infants with sufficiently dense measurements, which may not reflect all trajectories. Finally, multiple comparisons across vessels, parameters, and time windows raise the possibility of type I error; therefore, effect sizes and intervals should be prioritized over isolated p-values. For example, using the Benjamini–Hochberg (BH) method for multiple comparison correction^22^ led to losing statistical significance.

Despite those limitations, our study offers several important strengths. This is one of the few studies to analyze Doppler flow patterns of both CA and SMA in a well-defined cohort of preterm neonates born before 29 weeks of gestation. Moreover, our study included time-series analysis of Doppler parameters leading up to NEC onset, offering valuable insight into the temporal relationship between altered splanchnic perfusion and disease development, an area seldom addressed in previous studies. Although the sample size was relatively small and the retrospective nature of the study may limit generalizability, our findings contribute meaningful data to the growing evidence supporting the role of impaired intestinal blood flow in NEC pathogenesis. Larger, multicenter prospective studies incorporating serial Doppler measurements, clinical risk factors, and biomarkers of intestinal injury may provide a more comprehensive understanding of NEC pathogenesis and improve early detection strategies.

## Conclusion

The findings of low CA PSV in the first week of life and a decrease in SMA PSV in the week before NEC diagnosis may be linked to an increased risk of NEC. These findings add to the growing body of evidence that abnormalities in splanchnic blood flow could contribute to NEC development. However, inconsistencies across studies highlight the need for larger, prospective research that standardizes Doppler assessment timing, patient populations, and clinical care. Future studies with larger sample sizes are essential to determine if Doppler flow measurements can reliably predict NEC and help guide early prevention efforts in high-risk preterm infants.

## Supplemental Material

**Supplemental Material -** Association between celiac and superior mesenteric arteries Doppler flow parameters and risk of necrotizing enterocolitis in preterm infants Supplemental Material for Association between celiac and superior mesenteric arteries Doppler flow parameters and risk of necrotizing enterocolitis in preterm infants by Raef Qeretli, Abdalkarim Alnajjar and Nadya Ben Fadel in Journal of Neonatal-Perinatal Medicine.

## Appendix

### Abbreviations

AGA: Appropriate for gestational age
AMD: Adjusted mean difference
BW: Birth weight
CA: Celiac artery
CI: Confidence interval
cm/s: Centimeters per second
EDV: End-diastolic velocity
GA: Gestational age
NEC: Necrotizing enterocolitis
NICU: Neonatal intensive care unit
PDA: Patent ductus arteriosus
PSV: Peak systolic velocity
RI: Resistive index
SMA: Superior mesenteric artery
US: Ultrasound
SD: Standard deviation.
