# Supplementary material for: Association between celiac and superior mesenteric arteries’ Doppler flow parameters and risk of necrotizing enterocolitis in preterm infants
Source: J Neonatal Perinatal Med. 2025 Sep 13;19(2):172–80. doi: 10.1177/19345798251377439 (PMC12833023; doi:10.1177/19345798251377439)
Supplement: Supplemental Material - Association between celiac and superior mesenteric arteries Doppler flow parameters and risk of necrotizing enterocolitis in preterm infants [file sj-pdf-1-npm-10.1177_19345798251377439.pdf]

Supplementary Materials

Supplementary Table 1. *Aortic Doppler Flow Parameters in NEC vs Control Infants (First Week of Life)*

	Aorta‡	
	Control (N=13)	NEC (N=14)
**PSV (cm/s)**		
Mean (SD)	41.5 (18.5)	35.2 (12.8)
**EDV (cm/s)**		
Mean (SD)	3.83 (4.52)	4.62 (3.22)
**RI**		
Mean (SD)	0.906 (0.111)	0.867 (0.101)
**Age at Echo Scan (days)**		
Mean (SD)	3.77 (1.30)	4.43 (1.83)
Median [Min, Max]	4.00 [2.00, 6.00]	4.50 [2.00, 7.00]

‡ No significant differences found in adjusted and unadjusted analyses.
